# Tick Bites Induce Anti-α-Gal Antibodies in Dogs

**DOI:** 10.3390/vaccines7030114

**Published:** 2019-09-15

**Authors:** Adnan Hodžić, Lourdes Mateos-Hernández, Michael Leschnik, Pilar Alberdi, Ryan O. M. Rego, Marinela Contreras, Margarita Villar, José de la Fuente, Alejandro Cabezas-Cruz, Georg Gerhard Duscher

**Affiliations:** 1 Department of Pathobiology, Institute of Parasitology, University of Veterinary Medicine Vienna, 1210 Vienna, Austria; adnan.hodzic@vetmeduni.ac.at; 2 SaBio, Instituto de Investigación de Recursos Cinegéticos, IREC-CSIC-UCLM-JCCM, 13005 Ciudad Real, Spain; lourdes.mateos@vet-alfort.fr (L.M.H.); Maria.Alberdi@uclm.es (P.A.); marinela.contreras@uclm.es (M.C.); MargaritaM.Villar@uclm.es (M.V.); josedejesus.fuente@uclm.es (J.d.l.F.); 3 UMR BIPAR, INRA, ANSES, Ecole Nationale Vétérinaire d’Alfort, Université Paris-Est, 14 rue Pierre et Marie Curie, 94706 Maisons-Alfort, France; 4 Department for Companion Animals, Small Animal Clinic, University of Veterinary Medicine Vienna, 1210 Vienna, Austria; michael.leschnik@vetmeduni.ac.at; 5 Institute of Parasitology, Biology Center, Czech Academy of Sciences, 37005 České Budějovice, Czech Republic; ryanrego@paru.cas.cz; 6 Department of Veterinary Pathobiology, Center for Veterinary Health Sciences, Oklahoma State University, Stillwater, OK 74078, USA

**Keywords:** α-Gal, immune response, dog, tick bite, *Ixodes ricinus*, pathogens

## Abstract

Due to the functional inactivation of the gene encoding for the enzyme that is involved in the oligosaccharide galactose-α-1,3-galactose (α-Gal) synthesis, humans and Old-World primates are able to produce a large amount of antibodies against the glycan epitope. Apart from being involved in the hyperacute organ rejection in humans, anti-α-Gal antibodies have shown a protective effect against some pathogenic agents and an implication in the recently recognized tick-induced mammalian meat allergy. Conversely, non-primate mammals, including dogs, have the ability to synthetize α-Gal and, thus, their immune system is not expected to naturally generate the antibodies toward this self-antigen molecule. However, in the current study, we detected specific IgG, IgM, and IgE antibodies to α-Gal in sera of clinically healthy dogs by an indirect enzyme-linked immunosorbent assay (ELISA) for the first time. Furthermore, in a tick infestation experiment, we showed that bites of *Ixodes ricinus* induce the immune response to α-Gal in dogs and that the resulting antibodies (IgM) might be protective against *Anaplasma phagocytophilum*. These findings may help lead to a better understanding of the underlying mechanisms involved in mammalian meat allergy and tick-host-pathogen interactions, but they also open up the question about the possibility that dogs could develop an allergy to mammalian meat after tick bites, similar to that in humans.

## 1. Introduction

Galactose-α-1,3-galactose (α-Gal) is an oligosaccharide abundantly expressed on glycoproteins and glycolipids of non-primate mammals, prosimians, and New World monkeys. In contrast, humans and our primate ancestors lost the ability to synthetize the epitope due to functional inactivation of the enzyme α-1,3-galactosyltransferase (α1,3GalT) [1]. As a result of this unique evolutionary event that occurred about 28 million years ago, all immunocompetent crown catarrhines are able to produce a large quantity of antibodies (Abs) that specifically bind to the α-Gal epitope [2,3]. In general, the immune response to α-Gal can be classified as either beneficial or detrimental and the outcome largely depends on the source of the glycan molecule [4,5]. For instance, continuous exposure to α-Gal-containing bacteria of the normal intestinal flora leads to a production of anti-α-Gal antibodies (predominantly IgG and IgM), which were found to be protective against vector-borne and non-vector-borne pathogens carrying α-Gal on their surface [6,7]. These antibodies are also involved in the hyperacute rejection of xeno-transplants in humans [8]. More recently, production of IgE Abs induced by α-Gal from tick saliva has been linked to a delayed and life-threatening anaphylactic reaction to mammalian meat in human patients previously exposed to tick bites [9,10,11]. However, the epitopes in mammalian meat are exposed but unable to induce an anti-α-Gal IgE response until the individual is bitten by a tick, which suggests that certain tick salivary antigen(s) may break the peripheral food tolerance and trigger the immune complex reaction [4]. The strong correlation between this novel type of food allergy and tick bites has been demonstrated further by detecting glycoproteins with terminal α-Gal moieties in the saliva of several tick species, including *Ixodes scapularis* and *Ixodes ricinus* [12,13]. As in other non-primate mammals, α-Gal in dogs is expressed as a self-antigen and, thus, their immune system is not expected to naturally generate Abs toward this glycan molecule [1]. However, the results of this preliminary study demonstrated the specific immune response to α-Gal in dogs and suggested its possible relation to tick bites, and protection against tick-borne pathogens.

## 2. Materials and Methods

### 2.1. Dog Sera

Serum samples from 85 clinically healthy dogs from a previous study [14] were used in the present one. Essentially, the dogs were naturally exposed to ticks and the serum samples had been tested for Ab responses against three tick-borne pathogens affecting dogs in Austria, and these include *Anaplasma phagocytophilum*, *Borrelia burgorferi* sensu lato (s.l.), and tick-borne encephalitis virus (TBEV). For details on the sampling and pathogens’ diagnostic procedures, please see Leschnik et al. [14].

### 2.2. Antigen Preparation from Tick Salivary Glands

Salivary gland proteins (SGP) were extracted from unfed, adult female *I. ricinus* and *I. scapularis* ticks, obtained from a laboratory colony maintained at Biologie Moléculaire et Immunologie Parasitaire et Fongique (BIPAR, Maisons-Alfort, France) and the Oklahoma State University Tick Rearing Facility (Stillwater, OK, USA), respectively, and used as antigens for indirect enzyme-linked immunosorbent assay (ELISA). Salivary glands were retrieved after tick dissection and homogenized in lysis buffer containing 7M Urea, 2M Thiourea, 2% 3- ((3-cholamidopropyl) dimethylammonio)-1-propanesulfonate, CHAPS), or TRI Reagent™ Solution (Thermo Fisher Scientific, Waltham, MO, USA) with supplementation of a protease inhibitor cocktail (Roche, Basel, Switzerland). The homogenate was incubated in a thermomixer for 1 h at 20 °C with shaking at 800 rpm, and then centrifuged at 200× *g* for 5 min to remove cellular debris. The resulting supernatant was collected and the protein concentration was determined by a Bradford assay using bovine serum albumin (BSA, Sigma-Aldrich, St. Louis, MI, USA) as a standard [15].

### 2.3. Indirect ELISA

To evaluate levels of specific Abs in dog sera, 96-well ELISA plates (Nunc-Immuno^TM^ Plate, Roskilde, Denmark) were coated overnight at 4 °C with 100 µL/well of either Galα1-3Gal linked to human serum albumin (HSA) (0.5 µg/mL, Dextra Laboratories, Reading, UK) or soluble SGP derived from *I. ricinus* (0.5 µg/mL) and *I. scapularis* (0.5 µg/mL) ticks. The antigens were diluted in carbonate/bicarbonate buffer (0.05 M, pH 9.6). Optimal antigen concentration and dilutions of sera and conjugate were defined using a titration assay. The wells were washed three times with 150 µL of phosphate-buffered saline (PBS) containing 0.05% Tween 20 (PBS-T) and then blocked with 1% HSA (Sigma-Aldrich, USA) in PBS-T for 1 h at 37 °C. After five washes, serum samples, diluted in 0.5% HSA/PBS-T (1:800 for IgG, 1:400 for IgM and 1:10 for IgE), were added to the respective wells and incubated for 1 h at 37 °C. The plates were washed five times and horseradish-peroxidase (HRP)-conjugated Abs (sheep anti-dog IgG, goat anti-dog IgM, and goat anti-dog IgE, Bio-Rad, Rüdigheim, Germany) were added at 1:10,000 dilution in 0.5% HSA/PBS-T and incubated for 1 h at 37 °C. Lastly, the plates were washed five times and the reaction was developed by adding 100 µL ready-to-use tetramethylbenzidine-hydrogen peroxide (TMB) solution (Thermo Fisher Scientific, USA) at room temperature (RT) for 20 min in the dark, and then stopped with 50 µL of 0.5 M H_2_SO_4_. Optical densities (OD) were measured at 450 nm using an ELISA plate reader (Filter-Max F5, Molecular Devices, San Jose, CA, USA). All samples were tested in duplicate and the average value of four blanks (no serum) was subtracted from the reads. The cut-off was determined as two times of a mean OD value of the blank controls [16]. A monoclonal mouse anti-α-Gal antibody (mAb) M86 (Enzo Life Science Inc, Farmingdale, NY, USA), at dilution 1:100, was used as a positive control and HRP-goat anti-mouse IgM (diluted 1:4,000, Bio-Rad, Germany) as a secondary Ab.

In order to test the affinity of canine anti-α-Gal Abs to structurally different α-Gal epitopes, some dog sera were additionally tested against the Galα1-3Galβ1-4GlcNAc-HSA trisaccharide (Dextra Laboratories, Reading, UK).

The ratio between Abs against Galα1-3Gal-HSA and tick SGP was calculated using the following formula.

× = (anti-α-Gal/anti-tick SGP) × 100(1)

### 2.4. Inhibition ELISA

Wild type (WT) and alpha-1,3-galactosyltransferase (*ggta1*) knockout (KO) *Sus scrofa* kidney samples were individually placed in lysis buffer and homogenized with glass beads at 6000 rpm for 30 s using the homogenizer Precellys 24 Dual. The homogenization protocol was repeated 3 times. The lysate was then centrifuged at 10,000× *g* for 5 min to remove cellular debris. The supernatant was collected and the protein concentration was determined by a Bradford assay using BSA.

To evaluate the specificity of anti-α-Gal antibodies in dog sera, 96-well ELISA plates (Nunc-Immuno^TM^ Plate, Roskilde, Denmark) were coated overnight at 4 °C with 100 µL per well with WT or KO pig kidney proteins (0.5 µg/mL), diluted in carbonate/bicarbonate buffer (0.05 M, pH 9.6). The wells were washed three times with 150 µL of PBS containing 0.05% Tween 20 (PBS-T) and then blocked with 0.5% HSA (Sigma-Aldrich, USA) in PBS-T for 1 h at RT. Five dog sera diluted 1:400 in PBS and the mAb M86 antibody diluted 1:200 were preincubated overnight with 300 µg of WT or KO pig kidney proteins at 4 °C and constant shaking at 300 rpm. The protein-anti-α-Gal Abs complexes were removed by centrifugation at 16,000× *g* for 30 min at 4 °C. The supernatant was then collected and added to the wells for 1 h at 37 °C. The plates were washed five times and HRP-conjugated goat anti-dog IgM Ab diluted 1:10,000 in 0.5% HSA/PBS-T or HRP-conjugated goat anti-mouse IgM Ab 1:2000 were used as secondary antibodies for dog sera and M86 and incubated for 1 h at RT. Lastly, the plates were washed, the reaction developed, and the optical densities measured as described above. All samples were tested in triplicate and the average value of four blanks (no serum) was subtracted from the reads.

### 2.5. Enzymatic Removal of α-Gal to Test the Specificity of Canine Antibodies

To assess the specificity of immune responses to α-Gal in dogs, the Galα1-3Gal-HSA antigen was immobilized on an ELISA plate (50 ng/well), and then it was treated with α-galactosidase from green coffee beans (Sigma-Aldrich, USA) following the procedure described elsewhere [17]. Prior to the treatment, the enzyme was centrifuged at 10,000× *g* for 10 min at 4 °C, according to the manufacturer’s instructions to remove the ammonium sulfate. The supernatant was discarded and 100 mM potassium phosphate buffer (pH 6.5) was added to the pellet so the final concentration of the enzyme solution was 50 mU/100 µL. The plate was then incubated at 37 °C for 24 h in a humidified plastic chamber to avoid evaporation. After the incubation, wells were washed five times with 150 µL of PBS-T and the indirect ELISA was performed as described above. The enzymatic removal of the terminal α-Gal moieties from SGP of both *Ixodes* tick species was also performed following the same procedure. Ten individual and randomly selected dog sera were tested and the mAb M86 served as a positive control.

### 2.6. Experimental Infestation of Dogs with Ixodes ricinus

In order to investigate the ability of ticks to elicit a canine immune response to α-Gal, we experimentally infested dogs with larvae and adult *I. ricinus* ticks and compared the Ab levels before tick exposure and 39 days after the tick exposure by indirect ELISA. The *I. ricinus* were originally obtained from the reference laboratory colony maintained at the tick rearing facility of the Institute of Parasitology (Biology Center, Academy of Sciences of the Czech Republic) [18]. Three mixed breed male and female dogs (age > 6 months, body weight > 2 kg) were used for tick infestation. Each dog was infested with 55 adults (25 females and 30 males) of at least 10 days after molting and 100 larvae of approximately 15 days after hatching and placed in feeding chambers (one chamber per tick stage) glued on each dog’s shaved flank.

### 2.7. In Vitro Culture of IRE/CTVM20 Tick Cells and Human HL-60 Undifferentiated Promyelocytic Cells

*I. ricinus* embryo-derived cell line IRE/CTVM20 [19] and human HL-60 cells were maintained in L-15/L-15B and RPMI 1640 medium, respectively, as previously reported [20,21,22]. IRE/CTVM20 and HL-60 cells were used as positive and negative controls, respectively, of α-Gal expression [12].

### 2.8. Borrelia burgdorferi Culture

*B. burgdorferi* strains N40, B31, and JD1 were used for this study. Spirochetes were grown in BSK II supplemented with 6% rabbit serum (Sigma-Aldrich, Hamburg, Germany) at 34 °C to a density of 10^8^/mL [23]. Eight ml of this culture was then pelleted down by centrifuging at 7000× *g* for 10 min at RT.

### 2.9. Anaplasma phagocytophilum Culture and Purification

HL-60 cells were used to propagate the human isolate of *A. phagocytophilum* strain NY-18, as described previously [22]. Purification of *A. phagocytophilum* was done following the protocol published by Lis et al. [24].

### 2.10. Bacterial Preparation for Flow Cytometry

After dispensing the supernatant, the bacterial pellets were washed twice in PBS and, each time, they were centrifuged at 4000× *g* for 5 min at 4 °C. The cells were then fixed by adding 200 μL of 4% PFA in PBS to the pellet and incubated for 30 min at RT. The bacterial pellet was then washed once with PBS at 4000× *g* for 5 min at RT. One ml of PBS was added to the bacterial pellet and stored at 4 °C until further use.

### 2.11. α-Gal Detection by Flow Cytometry and Immunofluorescence in Human, Tick, and Bacterial Cells

α-Gal detection was performed as previously described [12]. Briefly, human, tick, and bacterial cells were washed in PBS, then fixed, and permeabilized. The cells were then incubated with 3% HSA (Sigma-Aldrich, USA) in PBS for 1 h at RT. Then, the cells were incubated for 14 h at 4 °C with the mAb M86 diluted 1:50. The viable cell population was gated according to forward-scatter and side-scatter parameters. FITC-goat anti-mouse IgM (Abcam, Cambridge, UK) labelled Ab (diluted 1:200 in 3% HSA/PBS, 1 h at RT) was used as a secondary Ab. The mean fluorescence intensity (MFI) was determined by flow cytometry and compared between the test and control cells by Student’s *t*-test with unequal variance (*p* < 0.05, *n* = four biological replicates). Aliquots of fixed and stained samples were used for immunofluorescence assays, mounted in ProLong Antifade with a DAPI reagent (Molecular Probes, Eugene, OR, USA) and examined using a Zeiss LSM 800 laser scanning confocal microscope (Carl Zeiss, Oberkochen, Germany) with oil immersion objectives.

### 2.12. Statistical Analyses

All data were statistically analyzed using GraphPad 5 Prism software (GraphPad Software Inc., San Diego, CA, USA). Nonparametric Kruskal-Wallis analysis with Dunn’s post-test was employed to evaluate the statistical significance of the specific Ab levels in relation to different antigens. A comparison between infected and uninfected dogs was done using the Mann-Whitney *U* test, while the difference in reactivity of the specific Abs before and after the enzymatic treatment was tested with the Wilcoxon matched pairs test.

Outliers were identified with Grubb’s test using an online calculator (https://www.graphpad.com/quickcalcs/Grubbs1.cfm) and removed from the final data. The correlation between immune responses to α-Gal and tick SGP was evaluated with the non-parametric Spearman test. Differences were considered significant if *p* < 0.05.

### 2.13. Ethical Statement

Experimental infestation of dogs with *I. ricinus* was conducted in strict accordance with the recommendations of the European Guide for the Care and Use of Laboratory Animals. Animals were housed and experiments were conducted at the LLC ACRO Vet Lab (Pylipovichi village, Kiev region, Ukraine) with the approval and supervision of the Ukrainian Commission for Bioethics and Biosafety for animals under the study “Tick vaccine experiments on dogs” number 000576.

## 3. Results and Discussion

### 3.1. Detection of Antibodies to α-Gal and the Specificity of the Immune Responses to the Glycan Epitope in Dogs

**Figure 1:**
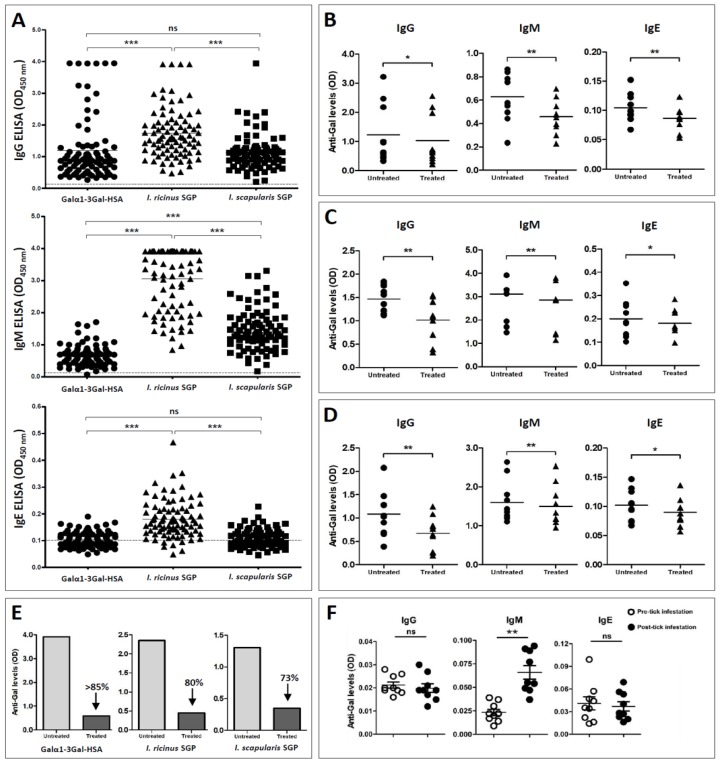
Immune responses to α-Gal and tick salivary gland proteins (SGP) in dogs naturally and experimentally exposed to ticks. (**A**) Distribution of canine IgG, IgM, and IgE serum antibodies against α-Gal, *I. ricinus* SGP, and *I. scapularis* SGP observed by ELISA (OD450 nm). The assay cut-off value for each isotype is indicated by the dashed line. The Nonparametric Kruskal-Wallis test was used to assess statistical significance: ns—not significant, *** *p* < 0.0001. (**B**–**E**) The specificity of canine anti-α-Gal tested by indirect ELISA. In comparison with the untreated group, a reduction in reactivity against (**B**) Galα1-3Gal-HSA, (**C**) *I. ricinus* SGP, and (**D**) *I. scapularis* SGP was observed after the antigen was pretreated with α-galactosidase. Ten individual and randomly selected dog sera were used as primary antibodies. (**E**) A substantial decrease in the reactivity after the enzymatic removal was also observed when the monoclonal anti-α-Gal antibody M86 was used as a positive control. Wilcoxon matched pairs test: * *p* < 0.05, ** *p* < 0.01. (**F**) Specific immune responses to α-Gal in three dogs experimentally exposed to *I. ricinus* (100 larvae and 55 adults) before the infestation and 39 days post-infestation. Results are representative of three technical replicates for each dog. Mann-Whitney U test: * *p* < 0.05, ** *p* < 0.01.

Serum IgG and IgM against α-Gal were detected in 85 (100%) and 84 (98.8%) animals, respectively, and almost half of the dogs (42/85, 49.4%) had detectable IgE to α-Gal (Figure 1A). The specificity of the reactivity of dog sera to α-Gal was tested (i) after enzymatic removal of terminal α-Gal residues from Galα1-3Gal-HSA and SGPs and (ii) after preincubation of dog sera with protein extracts from WT and α-Gal KO pigs in an ELISA inhibition assay. A significant decrease in the Ab reactivity after the enzymatic removal of terminal α-Gal residues from Galα1-3Gal-HSA antigen (Figure 1B) and SGP of *I. ricinus* (Figure 1C) and *I. scapularis* (Figure 1D) was observed. The same was seen when mAb M86 was used as a positive control (Figure 1E). These findings suggest the specificity of the canine immune response to α-Gal and further confirm the presence of α-Gal moieties in the saliva of both *Ixodes* tick species [25]. However, some dogs showed a strong background reactivity to BSA (but not to HSA) which, according to the user manual, is used as a stabilizing agent in the α-galactosidase enzyme solution.

**Figure 2:**
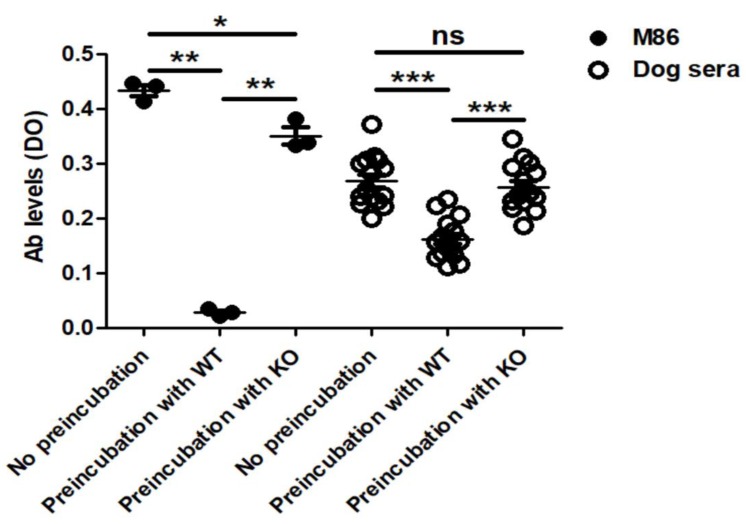
Reactivity of dog (*n* = 5) IgM serum Abs and M86 to wild type (WT) and α-Gal knockout (KO) pig proteins after a pre-incubation with WT proteins as measured in the ELISA inhibition assay. Paired *t*-test: ns—not significant, * *p* < 0.05, ** *p* < 0.01, and *** *p* < 0.0001.

The ELISA inhibition assay showed that pre-incubation of dog sera with WT, but not with KO protein extracts, decreased significantly the reaction to WT protein extracts compared to dog sera without pre-incubation (Figure 2). In addition, the reactivity of dog sera after pre-incubation with WT was lower than that after pre-incubation with KO. A similar trend was observed when mAb M86 was used as a positive control (Figure 2).

**Figure 3:**
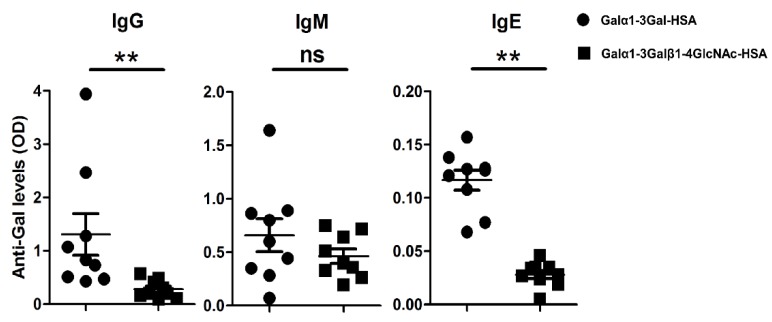
Reactivity of dog sera (*n* = 9) to Galα1-3Gal-HSA disaccharide and Galα1-3Galβ1-4GlcNAc-HSA trisaccharide as measured in indirect ELISA. Mann-Whitney *U* test: ns—not significant, ** *p* < 0.01.

The binding of natural Abs to α-Gal seems to be largely determined by the structural environment of the epitope, protein linker, and the number of terminal α-Gal epitopes [26,27]. Therefore, in the current study, the reactivity of the dog sera to α-Gal was tested against Galα1-3Gal disaccharide and Galα1-3Galβ1-4GlcNAc trisaccharide, coupled to HSA. The results showed that canine Abs recognized both epitopes, but the binding capacity was higher for Galα1-3Gal compared to Galα1-3Galβ1-4GlcNAc (Figure 3), which is in contrast to previous reports [28]. The affinity of the natural human anti-Gal Abs largely depends on a ‘pocket’ for the α-gal epitope with a size corresponding to the free trisaccharide Galα1-3Galβ1-4GlcNAc [28,29]. The trisaccharide is the most common form of the α-gal epitope in mammalian cells [28]. It is possible that the ‘pocket’ in canine anti-Gal Abs is smaller to that in human anti-Gal Abs and, therefore, canine anti-Gal Abs have lower affinity for the trisaccharide, which is expressed as a self-antigen in dogs.

The main hypothesis behind this intriguing discovery of anti-Gal Abs in dogs is that some components of tick saliva, in combination with α-Gal, modify the canine immune response, which leads to the induction of auto-Abs. More recently, Commins and colleagues [30] identified a possible enzyme candidate in the saliva of the Lone Star tick, *Amblyomma americanum*, called dipeptidyl-peptidase, which acts as an adjuvant. Furthermore, van Stijn et al. [31] observed a high level of IgG Abs against α-Gal when lambs were immunized with excretory/secretory antigens (ES) derived from the parasitic nematode *Haemonchus contortus* in Alhydrogel, known as an adjuvant that induces a strong T helper 2 (Th2) response. Given that both lambs and dogs abundantly express α-Gal epitopes on their cells [1], we hypothesize that a similar immunological reaction may also occur in dogs as a response to tick bites. Mimicry of host antigens, i.e., a molecular or epitope mimicry, is a mechanism widely used by parasites (presumably by ticks) and other infectious agents that enables them to evade the host immune system [32]. However, the similar antigenic properties of the two antigens may also induce an immune response, which breaks the tolerance to the host epitope and, subsequently, leads to autoimmune diseases [33]. Evidently, all the dogs included in the current study were clinically healthy and did not show signs of autoimmune diseases or any damaging effect of the anti-α-Gal Abs. The clear inability of the canine Abs to interact with self-Gal-antigens could be explained by an effective immune tolerance mechanism, similar to that occurring in people with the blood group B antigen [33]. Due to the structural similarity of the B antigen (Galα1-3(Fucαl,2)Gal) and α-Gal epitope, individuals with blood type A or O can produce anti-α-Gal Abs that also bind to the B antigen. However, the blood group B individuals have a reduced immune response to the epitopes since their immune system has been trained to ignore the self-antigen by producing less specific Ab clones [26,33].

### 3.2. Tick Bites Induce the Immune Response to α-Gal in Dogs

**Figure 4:**
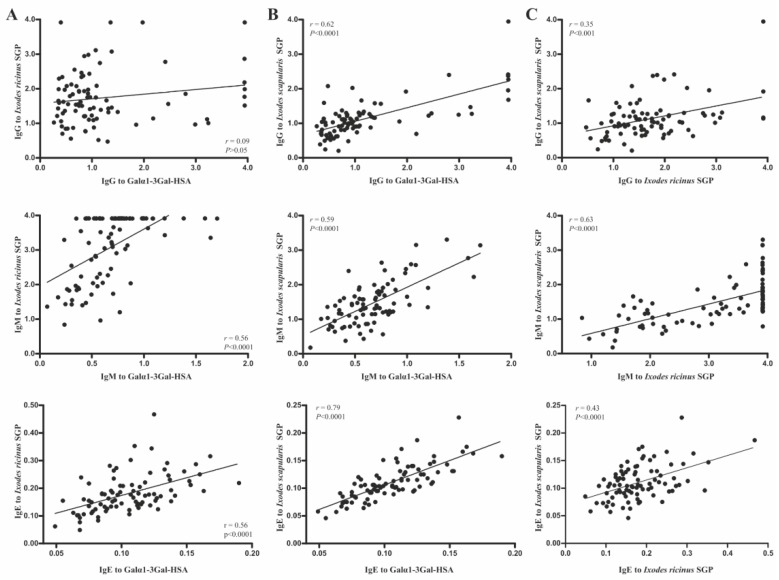
Correlations between antibody responses to α-Gal and SGP derived from (**A**) *I. ricinus* and (**B**) *I. scapularis*, and (**C**) between these two tick species using Spearman’s rank correlation coefficient (*r*).

To elucidate the possible relationships between α-Gal immune response and tick bites in dogs, sera were also tested for Abs against SGP from *I. ricinus* and *I. scapularis* (Figure 1A), which are common tick species in Europe and USA, respectively. The results revealed a strong correlation between IgM (*r* = 0.56, *p* < 0.0001) and IgE (*r* = 0.56, *p* < 0.0001) Ab levels to α-Gal and to *I. ricinus* SGP (Figure 4A), which suggests a possible role of this tick species in dog sensitization to α-Gal. In agreement with this result, a single experimental infestation with 100 larvae and 55 adult *I. ricinus* ticks increased the levels of IgM (*p* < 0.05) against α-Gal in dogs (Figure 1F). Despite this amount of exposure to ticks seeming extraordinarily high, it is common in dogs, but not in humans. The absence of an increase in IgE after tick infestation is in contrast with the previous studies, which report an association between IgE to α-Gal and IgE to *I. ricinus* (*r* = 0.58) and *A. americanum* (*r* = 0.75) in human patients with anaphylactic reactions to red meat [25,34]. However, it may be possible that repeated tick infestation, in opposition to a single one, are necessary to elicit anti-α-Gal IgE in dogs. IgG Abs to α-Gal and *I. ricinus* SGP did not correlate significantly (Figure 4A, *r* = 0.09, *p* > 0.05), which is in agreement with the fact that tick bites did not change the levels of IgG against α-Gal (*p* < 0.01) in experimentally-infested dogs (Figure 1F). This further suggests that anti-α-Gal IgG production, similarly to that in humans, is most likely related to the presence of gut microbiota or endoparasites expressing α-Gal epitopes [4,31], rather than to tick bites.

Even though the dogs were not exposed to *I. scapularis* (not existing in Europe), their sera recognized the tick epitopes and showed high correlations of anti-α-Gal IgG (*r* = 0.62, *p* < 0.0001), IgM (*r* = 0.59, *p* < 0.0001), and IgE (*r* = 0.79, *p* < 0.0001) Abs (Figure 4B). The Abs to both tick species displayed a positive and significant correlation between them (Figure 4C), which supports that *I. scapularis* and *I. ricinus* are sister taxa and share many salivary antigens. Furthermore, determination of the specific α-Gal/tick SGP ratios supports that the majority of epitopes in these two tick species are related to the anti-α-Gal response (Table 1).

### 3.3. Possible Implication of anti-α-Gal Antibodies in Protection Against Anaplasma phagocytophilum

**Figure 5:**
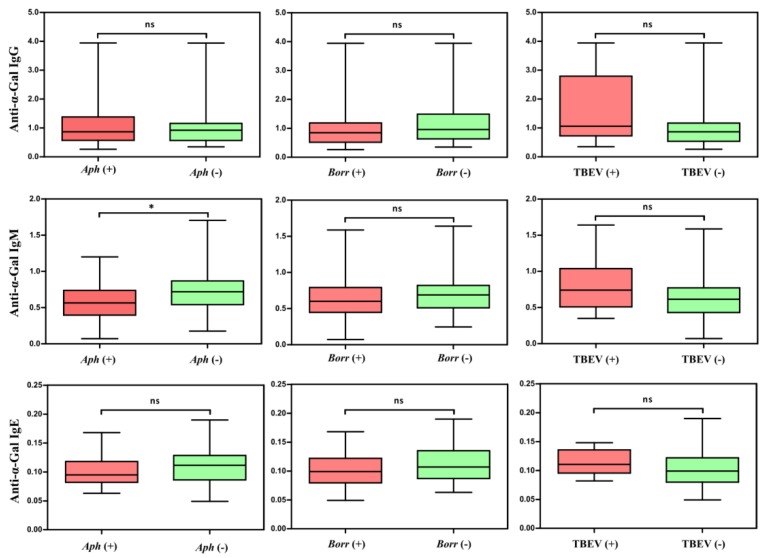
Relationships of IgG, IgM, and IgE antibodies to α-Gal between healthy dogs and dogs exposed to *Anaplasma phagocytophilum* (*Aph*), *Borrelia burgdorferi* sensu lato (*Borr*), and tick-borne encephalitis virus (TBEV). A Mann-Whitney *U* test was used to compare the difference between the groups: ns—not significant, * *p* < 0.05.

**Figure 6:**
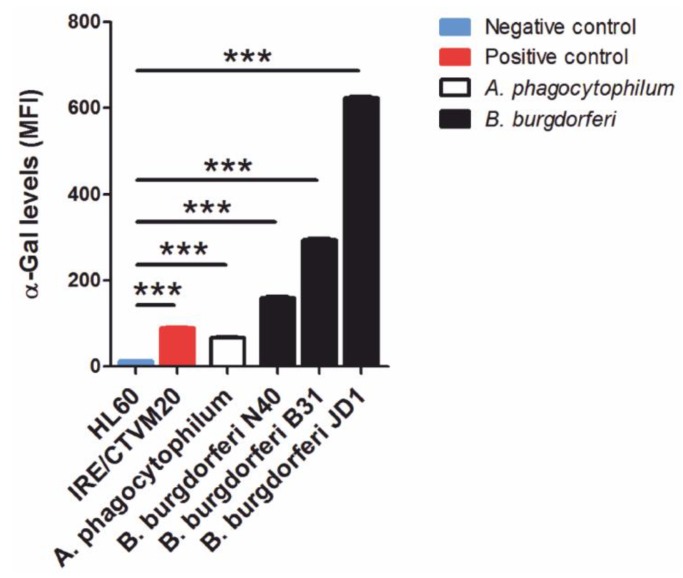
α-Gal levels in *Borrelia burgdorferi* sensu lato and *Anaplasma phagocytophilum*. α-Gal levels were measured by flow cytometry using the α-Gal-specific monoclonal antibody M86 (primary antibody) and the goat anti-mouse IgM-FITC antibody (secondary antibody). HL-60 and IRE/CTVM20 cells were used as negative and positive controls, respectively. Values in the axis *y* represent mean fluorescence values. Student’s *t*-test with unequal variance: *** *p* < 0.0001.

**Figure 7:**
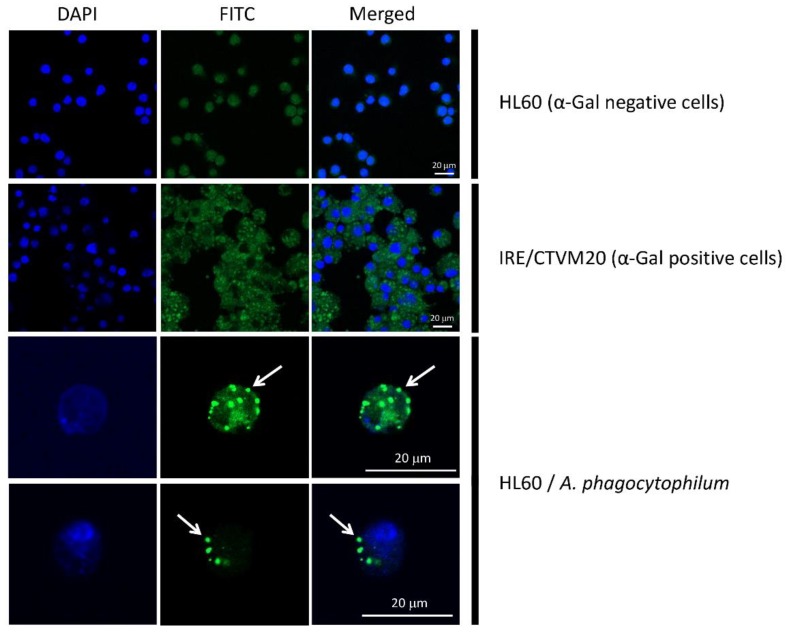
*Anaplasma phagocytophilum* infection is associated with the presence of α-Gal in α-Gal-negative cells HL-60. HL-60 cells were infected with *Anaplasma phagocytophilum* and α-Gal production was then measured by immunofluorescence. Uninfected HL-60 and tick cells IRE/CTVM20 were used as negative and positive controls, respectively. Cell nucleus was stained with DAPI (blue). The α-Gal-specific monoclonal antibody M86 (primary antibody) and the goat anti-mouse IgM-FITC antibody (secondary antibody) were used to detect the production of α-Gal (green). Merged images show that the presence of α-Gal was observed exclusively in the positive control and in *Anaplasma phagocytophilum*-infected HL-60 cells (arrows).

Lastly, the protective role of anti-α-Gal IgM and IgG against vector-borne pathogens (e.g., *Plasmodium* spp., *Trypanosoma* spp., and *Leishmania* spp.) containing this epitope on their surface has been well documented [7]. In this context, significantly higher levels of anti-α-Gal IgM were recorded in dogs negative to *A. phagocytophilum* infection (*p* < 0.05), which suggests the protective effect of this specific Ab isotype against anaplasmosis in dogs likely exposed to this pathogen (Figure 5). In contrast, no significant association was found between anti-α-Gal Abs and exposure to tick-borne encephalitis virus (TBEV) and *B. burgdorferi* (Figure 5). The presence of α-Gal was confirmed in *A. phagocytophilum* and *B. burgdorferi* s. l. by flow cytometry (Figure 6) and, additionally, in *A. phagocytophilum* by immunofluorescence (Figure 7). The lack of association between anti-α-Gal Abs and TBEV infection is expected because, like other viruses (i.e., Dengue), TBEV is a pathogen without α-Gal, and, therefore, cannot be targeted by the immune response to α-Gal [35]. Concerning *B. burgdorferi* s. l., as observed in Figure 6, the variability of α-Gal content in different strains is very high. A possible explanation is that, as a result of the evolutionary ‘*arms race*’ between the host and pathogen, *B. burgdorferi* strains infecting dogs do not produce or produce very low levels of α-Gal, which avoids recognition by anti-α-Gal Abs.

## 4. Conclusions

Although preliminary due to the limited sample size, the results of this study demonstrate the occurrence of canine Abs against α-Gal and indicate that tick bites can sensitize dogs to α-Gal. The results also suggest a protective role for anti-α-Gal Abs against tick-borne pathogens such as *A. phagocytophilum* in dogs. Our findings open a completely new scientific perspective and represent a baseline for future studies, which may contribute to a better understanding of the mechanisms involved in the generation of anti-α-Gal Abs in non-primate mammals, development of mammalian meat allergy, and tick-host-pathogen interactions. Additionally, our results suggest that attention should be given to the possibility that anti-α-Gal Abs may help prevent canine vector-borne diseases, but this requires further experimental in vivo and in vitro studies. Lastly, investigating whether dogs, similar to humans, can develop allergic reactions to mammalian meat following tick bites and induction of specific IgE Abs, should be the focus of future research studies.

## Figures and Tables

**Table 1:** Specific ratios between α-Gal and tick SGP antibody immune response.

Antibody	α-Gal/*I. ricinus* SGP (%)	α-Gal/*I. scapularis* SGP (%)
IgG	80.7	95.3
IgM	22.7	49.6
IgE	63.3	97.1
